# Bar-Coded Pyrosequencing Reveals the Responses of PBDE-Degrading Microbial Communities to Electron Donor Amendments

**DOI:** 10.1371/journal.pone.0030439

**Published:** 2012-01-25

**Authors:** Meiying Xu, Xingjuan Chen, Mengde Qiu, Xiaowei Zeng, Jian Xu, Daiyong Deng, Guoping Sun, Xiang Li, Jun Guo

**Affiliations:** 1 Guangdong Provincial Key Laboratory of Microbial Culture Collection and Application, Guangdong Institute of Microbiology, Guangzhou, Guangdong, China; 2 Bioenergy Genome Center and Shandong Key Laboratory of Energy Genetics, Qingdao Institute of Bioenergy and Bioprocess Technology, CAS, Qingdao, Shandong, China; 3 State Key Laboratory of Applied Microbiology, Ministry—Guangdong Province Jointly Breeding Base, Guangzhou, Guangdong, China; 4 Guangdong Open Laboratory of Applied Microbiology, Guangzhou, Guangdong, China; 5 Key Laboratory of Marine Bio-resources Sustainable Utilization, South China Sea Institute of Oceanology, CAS, Guangzhou, Guangdong, China; Argonne National Laboratory, United States of America

## Abstract

Polybrominated diphenyl ethers (PBDEs) can be reductively degraded by microorganisms under anaerobic conditions. However, little is known about the effect of electron donors on microbial communities involved in PBDEs degradation. Here we employed 454 Titanium pyrosequencing to examine the phylogenetic diversity, composition, structure and dynamics of microbial communities from microcosms under the conditions of different electron donor amendments. The community structures in each of the five alternate electron donor enrichments were significantly shifted in comparison with those of the control microcosm. Commonly existing OTUs between the treatment and control consortia increased from 5 to 17 and more than 50% of OTUs increased around 13.7 to 186 times at least in one of the microcosms after 90-days enrichment. Although the microbial communities at different taxonomic levels were significantly changed by different environmental variable groups in redundancy analysis, significant correlations were observed between the microbial communities and PBDE congener profiles. The lesser-brominated PBDE congeners, tri-BDE congener (BDE-32) and hexa-BDE, were identified as the key factors shaping the microbial community structures at OTU level. Some rare populations, including the known dechlorinating bacterium, *Dehalobacter*, showed significant positive-correlation with the amounts of PBDE congeners in the consortia. The same results were also observed on some unclassified bacteria. These results suggest that PBDEs-degrading microbial communities can be successfully enriched, and their structures and compositions can be manipulated through adjusting the environmental parameters.

## Introduction

Polybrominated diphenyl ethers (PBDEs) have been integrated into common household and industrial appliances as flame retardants for more than three decades, and were estimated to be more than 67 million kilograms in annual global market demand [1]. As a result of the widespread use, PBDEs residues are found in a wide variety of environment, and their concentrations have increased exponentially [2]–[3]. Since increasing evidence shows that PBDEs are bioaccumulated and biomagnified, these compounds have been listed as the new Persistent Organic Pollutants (POPs), and their fate and transport in ecosystems have received worldwide attention.

Similar to polychlorinated biphenyls (PCBs) in structure and characteristics, PBDEs are hydrophobic, semi-volatile, toxic, resistant to microbial degradation, and strong adsorption on sediments [4]. Relatively high concentrations of PBDEs, ranged from 51.3 to 365 ng/g in sediments have been detected in Guiyu of Guangdong Province [5], and the local environment is seriously polluted by these compounds due to open burning and uncontrolled dumpling processes. De Boer et al. [6] observed that free thyroxin hormone levels in animals decreased after exposure to PBDEs, and others [7]–[8] found that some congeners have strong binding affinities to human estrogen receptors. Therefore, it is urgent to develop potential remediation technologies to remove, degrade, or immobilize PBDEs in heavy contaminated industrial sites.

Recent studies have shown that PBDEs may be reductively debrominated by microorganisms in sediments [9]–[10]. However, it is rather slow for these highly persistent compounds to be transformed under the anaerobic conditions in sediments. Tokarz et al. [10] reported that deca-BDE was debrominated to less brominated congeners after 3.5 years of incubation in anaerobic sediment microcosms. Electron donor amendments, which were performed to stimulate the activities of indigenous microbial communities, have been employed as promising strategies for the remediation of contaminated environments [11]–[13]. Lee and He [9] found that the amendment of trichloroethene (TCE) promoted the growth of *Dehalococcoides* species during the PBDEs debromination process. Our previous study also found that the amendment of electron donors (methanol, ethanol, acetate, lactate and pyruvate) substantially shifted the PBDE-degrading microbial community structure based on 16S rDNA DGGE analysis and the PBDE-degrading product profile [14]. However, due to the limited resolution of DGGE analysis, detailed information about the diversity, composition and structure of microbial communities from PBDE-degrading microcosms and their correlations with the environmental parameters remains elusive.

454 Titanium pyrosequencing of 16S rRNA genes has been developed as a high throughput metagenomic technology for profiling microbial communities [15], which generates one million bp reads with an average length of over 400 bp, providing rich information for bacterial identification in a resolution at the species/strain level. To better understand the effect of electron donors on the PBDE–degrading communities and develop PBDEs bioremediation strategies, this high throughput approach was used to evaluate the PBDE-degrading microbial communities in this study. Our results showed that diversities, compositions and structures of PBDE-degrading microbial communities shifted under different electron donor amendments, and such changes were closely related to the controlled environmental parameters. To our best knowledge, this is the first attempt to use pyrosequencing technology for profiling microbial communities involved in PBDEs transformation and degradation.

## Materials and Methods

### Microcosm establishment

The inoculum sediment for all enrichment cultures was collected from the riverside of Lianjiang at Guiyu, a town has been involved in e-waste “recycling” for approximately 10 years [16], and contained about 6 g total organic carbon (TOC)/kg. The location of the sampling site is not privately-owned and no specific permits were required for the field studies. Sediment samples were preserved at 4°C fridge until further analysis and applications. Microcosms were established aseptically in an anaerobic glovebox, in which 20 g sediments (wet weight) and 150 mL defined medium were dispersed into 250-mL serum bottles. The defined medium contained 5.7 mM Na_2_HPO_4_, 3.3 mM KH_2_PO_4_, 18.0 mM NH_4_Cl, vitamin solution and mineral solution [17], 0.2 g/L of yeast extract and 10 mM of electron donors, such as methanol, ethanol, acetate, lactate or pyruvate, respectively, were used as carbon and energy sources. Prior to the addition of sediments (wet weight) and defined medium, 10 µM of BDE-209 resolved in dichloromethane was added to each serum bottle and evaporated in the dark. All the sample bottles in triplicates were purged with pure nitrogen gas for 5–10 min, and incubated at 30°C in anaerobic glovebox without agitation in the dark. The microcosms were transferred to fresh medium following on PBDEs quantitative assay biweekly. During the experiments, control batches in triplicates were prepared in the same way as mentioned above, except that no electron donor was added.

### Chemical analyses

For quantitative assay on the changes of bromide ion concentration in the microcosms, 2 mL of the culture medium were taken biweekly. Before analysis, the samples were pretreated with Ion chromatograph (IC) (Dionex-ICS2000) equipped with AS19 column to remove organic matters and heavy metals. Quantifications of bromide were performed by establishing six-point calibration curves as described by Qiu et al. [14]. For quantitative assay on the PBDE-degrading product congeners, the samples were extracted by the mixture of acetone and hexane (1∶1) and analyzed by GC-MS according to the procedures described by Mai et al. [16]. Standard curves of PBDEs were prepared by diluting the stock solution of PBDEs standard with n-hexane to 0.01, 0.025, 0.075 and 0.125 ug/mL and analyzed with GC-ECD. Linear regression equations with *r^2^* were obtained by plotting the integration area (*y*) vs. PBDE concentration (*x*) for calculating.

### DNA extraction, PCR, and 454 Titanium pyrosequencing

Metagenomic DNA of each enriched microcosm was extracted using the TIANamp Bacteria DNA Kit (TIANGEN BIOTECH (BEIJING) CO., LTD.) according to the manufacturer's instructions. Fragments of 16S rRNA genes containing variable V4-V5 regions were amplified from the extracted DNA with primers 515F (5′ GTGCCAGCMGCCGCGG 3′) and 907R (5′ CCGTCAATTCMTTTRAGTTT 3′) with a unique 6-mer tag or barcode for each sample. Each PCR reaction was performed in two of 50 µl reaction mixtures containing 20 ng of DNA, 200 µM of dNTP, 0.4 µM of each primer, 1× Pyrobest buffer (TaKaRa), and 1.25 unit of Pyrobest DNA Polymerase (TaKaRa). Reactions were cycled with an initial denaturation at 94°C for 5 min; 25 cycles of 94°C for 30 s, 60°C for 30 s, and 72°C for 30 s; and a final extension at 72°C for 7 min. The amplicons were purified by gel electrophoresis/isolation and a QIAquick Gel extraction kit (Qiagen, CA, USA). Amplicon pyrosequencing was performed by a 454/Roche GS-FLX Titanium instrument (Roche, NJ, USA) according to standard protocols.

### Sequence analysis

Low-quality sequences were filtered using the Pipeline Initial Process provided on the Ribosomal Database Project website (http://rdp.cme.msu.edu/) to remove sequences shorter than 150 nucleotides and lower than 20 of an average quality score [18]. Multiple sequence alignment and complete linkage clustering were used to cluster the sequences from 0% to 10% dissimilarity using Mothur v.1.11.0 modified from Sogin et al. [19]. These clusters served as operational taxonomic units (OTUs) for generating rarefaction curves and for calculating the richness and diversity indexes. Representative sequences from each OTU were phylogenetically assigned with taxonomic classifications obtained from the RDP's Classifier 2.0 and Mothur v.1.11.0. To describe similarity and difference between the communities, OTUs that were shared between communities or unique to a community were obtained by Mothur v.1.11.0 [20].

### Statistical analysis

The pre-processed 454 pyrosequencing dataset containing the OTUs shared by at least two of five biological replicates was further analyzed with different statistical methods, including principal component analysis (PCA), detrended correspondence analysis (DCA) and cluster analysis (CA). CANOCO 4.5 (Biometris – Plant Research International, The Netherlands) was used for PCA and DCA based on the relative abundance of OTUs detected by 454 pyrosequencing. CA was performed based on the relative abundance of OTUs detected by 454 pyrosequencing using the pairwise complete-linkage hierarchical clustering algorithm [21] provided in the Gene Cluster software (http://rana.stanford.edu), and the resulting clusters were visualized using the TREEVIEW software (http://rana.stanford.edu/). Redundancy analysis (RDA) and partial-RDA were performed to link the microbial community structure to environmental parameters. The significant environmental variables (*p*<0.05) were selected using a forward selection procedure followed by a Monte Carlo Permutation test based on 999 random permutations of the residuals under the full regression model using the package CANOCO 4.5. To identify patterns of variation among microbial communities, environmental variables were normalized by subtraction of the mean and division by standard deviation before performing multivariable analyses. Significant Pearson's linear correlation (*r*) analysis was conducted in SPSS 16.0 for windows (SPSS Inc., Illinois, USA).

## Results

### Overview of pyrosequencing statistics

Pyrosequencing produced a total of 18,615 high-quality V6 tags of the 16S rRNA genes from a single lane of a 16-lane PicoTiterPlate on a 454 Titanium system and the numbers of sequences from different samples varied in the range of 2,134 to 7,112. All sequences were aligned using the RDP Infernal Aligner and the complete linkage clustering method was used to define OTUs using 97% sequence identity as a cutoff, resulting in 1235 OTUs for methanol treatment, 755 OTUs for ethanol, 871 OTUs for acetate, 2037 OTUs for lactate, 2187 OTUs for pyruvate and 1428 OTUs for control, respectively.

Based on the non-parametric richness indexes of Chao1, approximately 34.4% to 42.8% of the predicted OTUs were sampled with different electron donors as the following trend: ethanol<acetate<methanol<control<lactate<pyruvate. For Simpson's diversity index (*1/D*), the treatment with lactate had the highest diversity (157.33) following by, pyruvate (140.31), methanol (131.46), acetate (121.36), control (101.36), and ethanol (94.08). Shannon-Weaver index (*H′*) had the similar trend as Simpson's diversity index, except the close index for the treatments with lactate and pyruvate (Table 1).

**Table 1:** Comparison of phylotype coverage and diversity estimation of the 16S rRNA gene libraries from the pyrosequencing analysis.

Sample	Methanol	Ethanol	Acetate	Lactate	Pyruvate	Control
Reads	2905	2134	2191	5917	7112	4139
Phylum^a^	18	15	14	16	17	17
Class^a^	30	18	21	26	24	28
Order^a^	41	30	33	35	40	49
Family^a^	71	49	55	62	67	84
Genus^a^	111	85	83	99	107	124
OTU^a^	1235	755	871	2037	2187	1428
*1/D*	131.46	94.08	121.36	157.33	140.31	101.36
*H′*	9.02	8.49	8.74	9.43	9.45	8.96
Chao1	3590	1765	2328	4998	5546	4059
ACE	6678	3014	4502	8753	9333	7274

a The operational taxonomic units (OTU) were defined with 3% dissimilarity.

**Figure 1:**
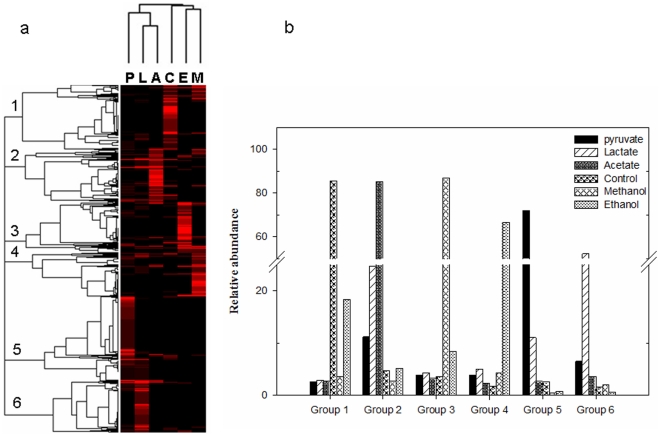
Hierarchical clustering analysis for microbial community profiles of the tested enriched microcosms based on all OTUs detected by barcoded pyrosequencing. The OTUs were defined with 3% dissimilarity. The figure was generated using Cluster and visualized in Treeview. The color scale indicates relative abundances. Six samples were clearly separated into two groups and six OTU patterns were observed and indicated by numbers in the tree (a), and also illustrated in the graphs (b). The abbreviations were represent the control microcosm without electron donor amendment (C), or enriched with methanol (M), ethanol (E), acetate (A), lactate (L), or pyruvate (P).

The unconstrained PCA was used to examine overall patterns of variation in microbial community structure with relative abundance of sequence data. The microcosms amended with acetate, lactate and pyruvate are grouped together and well separated from the other three microcosms along PC1, which explained 27.6% of the total variance (Figure S1). The microbial community profiles constructed by hierarchical clustering analysis showed significantly different patterns among the treatments (Figure 1a), which was coincided with the DGGE results [14]. Six major OTU groups can be visualized (Figure 1b), which were well correlate with the electron donor amendments. Group 1, 2, 3, 4, 5 and 6 in the color tree from up to down were mostly contributed by the populations from the control, acetate, methanol, ethanol, pyruvate, and lactate treatments with 73.9%, 63.8%, 78.7%, 79.4%, 80.3% and 78.3% total abundance, respectively. *Thiobacillus* spp. (12.1%) and *Comamonas* spp. (6.5%) from *Betaproteobacteria* were the most dominant populations in Group 1 and 5, *Pseudomonas* spp. (5.5%) and *Aminiphilus* spp. (8.4%) from *Gammaproteobacteria* in Group 2 and Group 3, while *Acholeplasma* spp. (6.5%) from class *Mollicutes* and *Synergistia* spp. (10.3%) from class *Synergistia* were the dominant populations in Group 4 and 6, respectively. These results suggest that the microbial community composition and structures are significantly altered by the electron donor amendments.

### Changes of microbial community structure in microcosms with alternate electron donors

#### (i) Changes at phylum level

**Figure 2:**
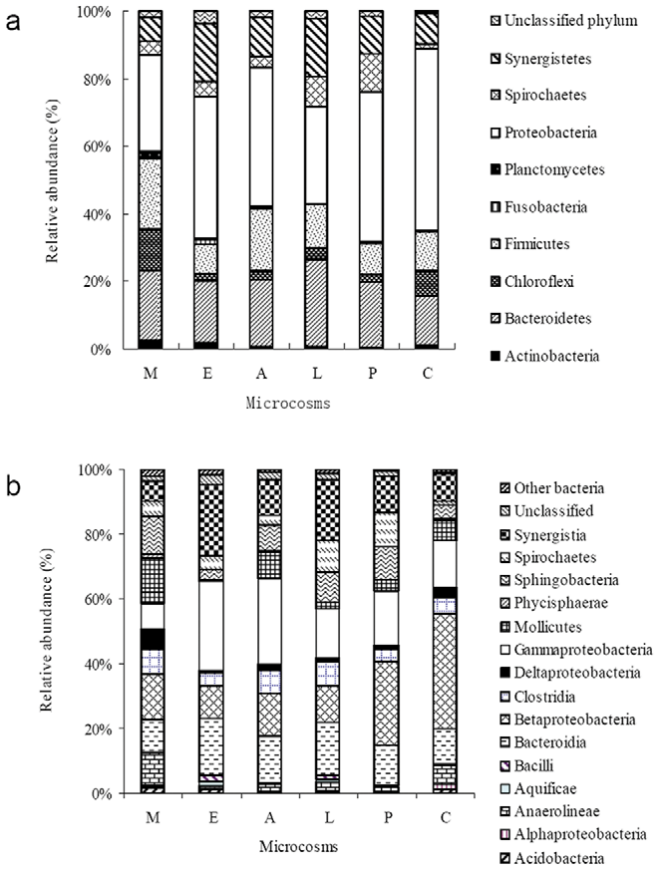
The predominant bacterial phyla (a) and classes (b) whose relative abundance were higher than 1.0% at least in one of the microcosms. The abbreviations were represent the control microcosm without electron donor amendment (C), or enriched with methanol (M), ethanol (E), acetate (A), lactate (L), or pyruvate (P).

The significant changes of microbial community structures in the microcosms with electron donor amendments were observed based on the microbial populations. Among the nine abundant phyla, whose relative abundances are higher than 1.0% at least in one of the microcosms, *Proteobacteria* was the dominant phylum in all of the treatments. However, the population of *Proteobacteria* was significantly decreased after electron donors were added (Figure 2a: W, E, A, L, and P). *Bacteroidetes* were detected as the second abundant phylum, and its population increased after the electron donors were added. The populations of chemoheterotrophic *Spirochaetes* phylum and the unclassified phyla detected also increased in the microcosms supplied with electron donors (Figure 2a).

#### (ii) Changes at class level

Among the 46 classes detected, 23 of them were commonly existed in all of the treatments and 15 of them were shown as the abundant classes (Figure 2b) with higher than 1.0% relative abundance at least in one of the microcosms. *Betaproteobacteria* and *Alphaproteobacteria* were ranked as the first and 14^th^ abundant class detected, and their populations decreased in the microcosms amended with electron donors. Meanwhile, the other three classes, *Spirochaetes*, *Verrucomicrobiae* and *Erysipelotrichi*, increased in the microcosms with additional electron donors. *Gammaproteobacteria*, *Bacteroidia*, *Synergistia*, and *Clostridia* were the predominant classes following *Betaproteobacteria*, but no obvious trend was observed among the treatments (Figure 2b). Within the six unique classes detected, two were from the microcosm enriched with methanol, and the others were from the microcosms with ethanol, acetate, lactate and control as well. Most of the unique classes were low abundant (<1%), including the class *Dehalococcoidetes* from methanol-amended microcosm.

#### (iii) Changes at genus level

**Figure 3:**
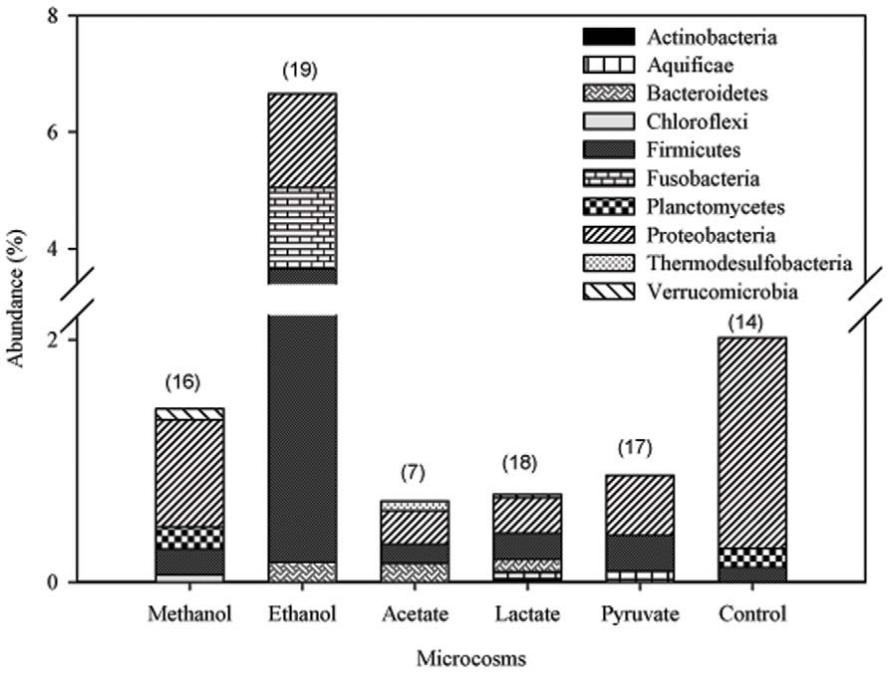
The abundances of the unique genera detected in bacterial phyla in the defined microcosm. The number of the unique genera detected was presented above the bars in parentheses.

Among the 271 genera detected, 91 genera were unique to the defined microcosms with 16 to the microcosm enriched with methanol, 19 to ethanol, 7 to acetate, 18 to lactate, 17 to pyruvate and 14 to the control. All of these unique genera belonged to 10 phyla and *Proteobacteria* was shown as the most dominant phylum in all microcosms, except the one enriched with ethanol, in which *Firmicutes* was the most dominant phylum (Figure 3). Although more than 58% of the unique genera were only detected from one OTU and most of them with quite low relative abundance (<0.1%), some of them have been isolated and reported as the debrominating bacteria, such as *Lysinibacillus* spp. [22].

**Figure 4:**
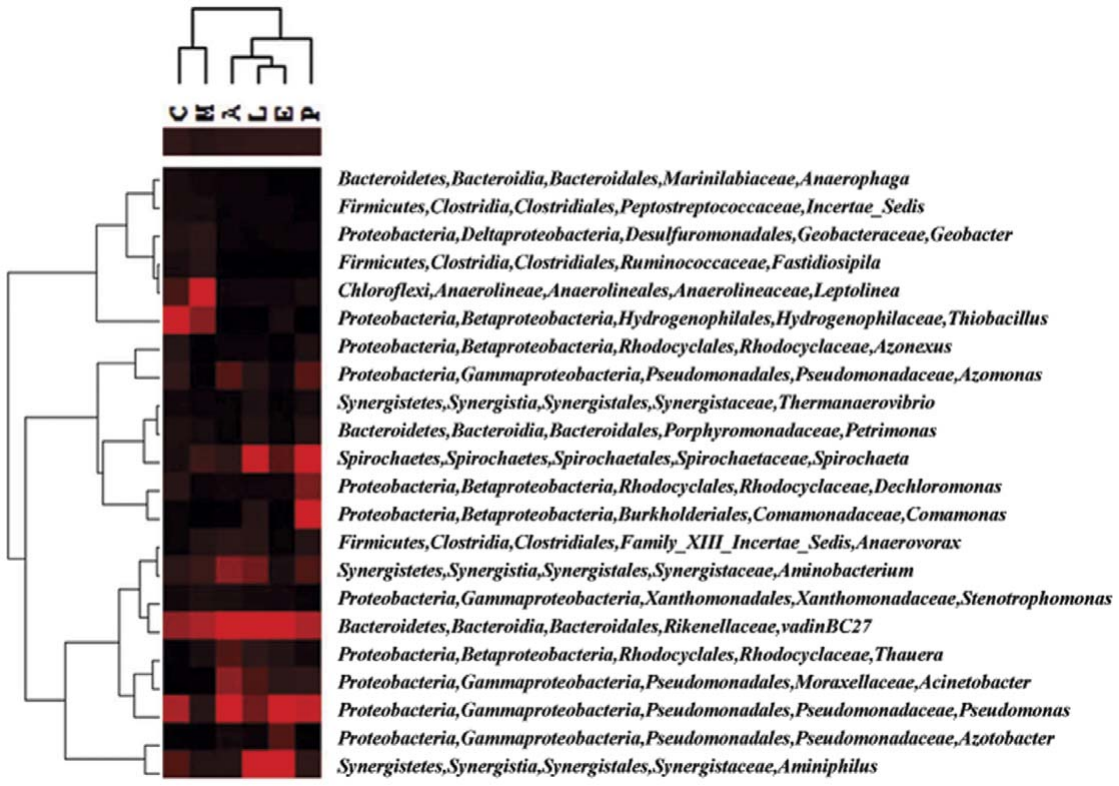
The predominant bacterial genera existing in common whose relative abundances were higher than 1.0% at least in one of the microcosms. The abbreviations were represent the control microcosm without electron donor amendment (C), or enriched with methanol (M), ethanol (E), acetate (A), lactate (L), or pyruvate (P).

On the other hand, 41 out of the 271 genera detected were commonly present in all six microcosms with 22 genera as predominant bacterial genera, whose relative abundances were higher than 1.0% at least in one of the microcosms (Figure 4). Within these 22 commonly existed, predominant bacterial genera, the most predominant genus detected was closed with the unidentified eubacterial clone vadinBC27, and its abundance significantly increased in the microcosms amended with electron donors. This genus was found as the dominant anaerobic digesters in a fluidized-bed reactor fed by vinasses [23]. The members of *Pseudomonas* and *Aminiphilus* were highly enriched in almost all of those microcosms, especially the amendment with ethanol. The abundances of genera *Spirochaeta*, *Acinetobacter* and *Thauera* significantly increased, while the abundances of genera *Thiobacillus*, *Azonexus* and *Anaerophaga* significantly decreased with electron donor amendments.

The known dechlorinating bacteria, *Dehalococcoides*, *Dehalobacter*, and *Desulfuromonas*, were only detected from one or two of the consortia in low abundances. *Dehalococcoides* was only detected in the methanol-amended microcosm with 0.15% relative abundance, and *Dehalobacter* was detected in methanol- and ethanol-amended microcosms with 0.30% and 0.50% relative abundances, respectively, while *Desulfuromonas* [24] was detected in the microcosms with lactate amendment and control with 0.01% and 0.06% relative abundances, respectively.

#### (iv) Changes in bacterial OTUs

**Figure 5:**
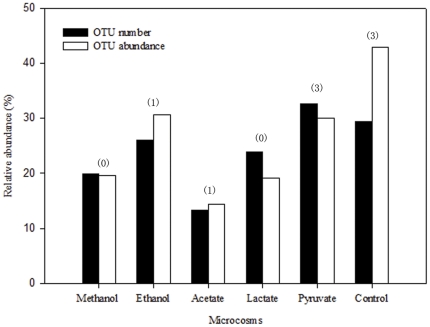
The percentages of the unique OTU number and relative abundance to the total OTU number and abundance detected in the defined microcosm. The numbers of the predominant OTUs whose relative abundance were higher than 1.0% were presented above the bars in parentheses.

Among a total of 4861 OTUs detected,2216 OTUs were only present in one microcosm, and their relative abundances were 19.57%, 30.65%, 14.39%, 19.16%, 29.95%, and 42.96% in the microcosms enriched with methanol, ethanol, acetate, lactate, pyruvate and control treatment, respectively (Figure 5). Individually, almost all of the unique OTUs were low-abundant (<1%), except eight OTUs (OTU7760, 9046, 13154, 13306, 13339, 16181, 16208, 16218) which all belonged to the commonly existing genera. OTU7760, which is closely relative with known anaerobic amino-degrading bacterium *Aminiphilus*, was unique to ethanol treatment with 4.18% relative abundance. OTU13306 (1.26%), OTU13154 (1.17%) and OTU13339 (1.13%) were unique in pyruvate treatment and closely relative with the main aromatic compound oxidizing contributors, *Dechloromonas* [25], *Comamonas* [26] and *Acinetobacter* [27], respectively. In the control microcosm, the first and second predominant unique OTUs, OTU16181 and OTU16218, were identified as *Thiobacillus* spp. and with 7.83% and 1.32% relative abundances, respectively, the third predominant unique OUT (OTU16208) were identified as *Dechloromonas* spp. and with 1.16% relative abundances. *Thiobacillus* species are the most commonly identified microorganisms in environments containing sulfur and heavy metals, such as acid mine drainage, and triggers critical organic soil mineral improvement and heavy metal remediation immediately [28]. *Dechloromonas* was reported to be able to reduce chlorate and perchlorate [29], even degrade aromatics with chlorate or perchlorate as a suitable electron acceptor [25]. OTU9046 (1.76%), which is closely relative with anaerobic protein-utilizing bacterium *Proteiniborus* [30], was unique in acetate treatment. The relative abundances of the unique OTUs detected in methanol and lactate treatments were smaller than 1%.

**Figure 6:**
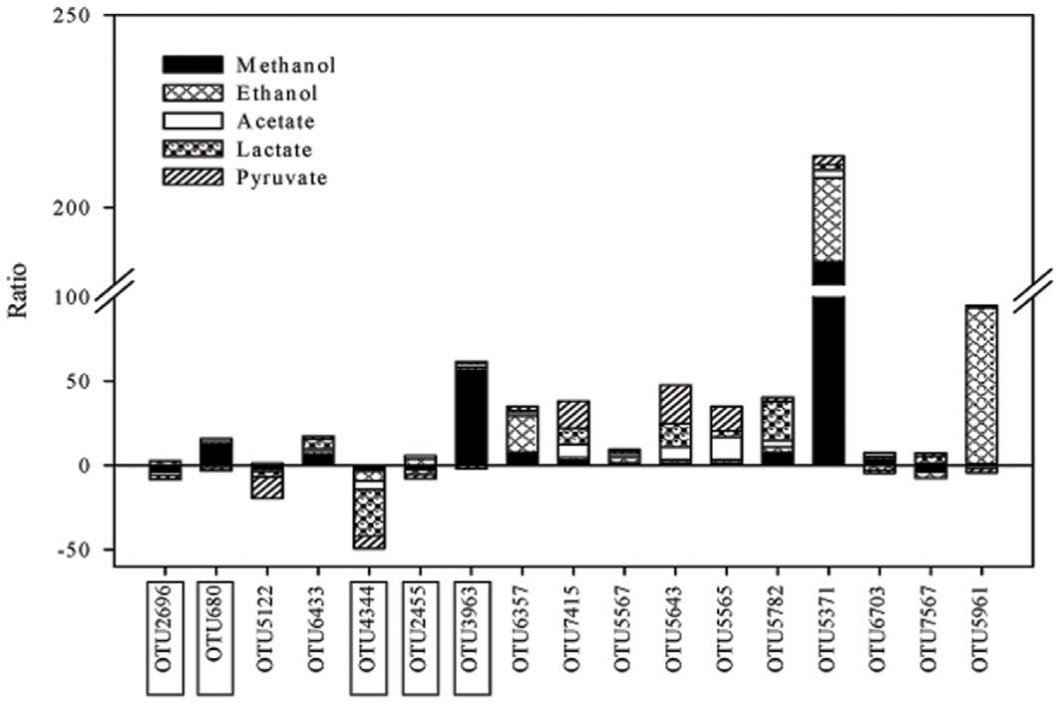
Commonly existing OTUs among the treatments with different electron donor amendments. Ratios were the quotient of the high relative abundance dividing the low relative abundance from treatment or control. Positive ratio means the relative abundance increased in the treatments with electron donors, while the negative ratio means decrease. The labels with rectangle were present in the initial inocula.

Furthermore, 17 OTUs were detected as the common populations in all of the six enriched consortia and only five of them (OTU2696, 680, 4344, 2455, 3963) were determined in the initial inoculum sediment (Figure 6). Most of those common OTUs may have been rare populations in the enriched community since their relative abundances were all smaller than 1%, except OTU3963 and OTU5371 with 1.13% and 3.71% for methanol consortia, and OTU5961 with 5.55% for ethanol consortia, respectively. OTU3963 was closely relative with known anaerobic fermenters *Leptolinea* sp. isolated from sludge granules of mesophilic UASB reactors treating sugar-processing wastewater [31], OTU5371 was identified as the unclassified *Sphingobacteriales* WCHB1–69 clone which was initially obtained from a hydrocarbon- and chlorinated-solvent-contaminated aquifer [32], and OTU5961 was identified as the uncultured anaerobic digester vadinBC27 [23]. Within these 17 common OTUs, eight OTUs (OTU5371, 5565, 5567, 5643, 5782, 6357, 6433, 7415) were commonly enriched when electron donors were supplemented, and nine OTUs (OTU680, 3963, 5371, 5565, 5643, 5782, 5961, 6357, 7415) were enriched more than 10 times at least in one of the microcosms. The abundance of OTU5371 increased in all electron-donor-added microcosms with around 186 and 22 times enriched in methanol- and ethanol-added microcosms, respectively. OTU5961 was enriched around 93 times in the methanol-added microcosm, but depressed in the microcosms supplied with acetate and pyruvate (Figure 6). The three OTUs, OTU5565 belonged to *Stenotrophomonas* sp., OTU6357 and OTU7415 belonged to *Pseudomonas* spp., were all enriched with electron donor amendment. OTU 4344, which was present in the initial inocula and identified as *Dechloromonas*, was minor population (0.01%-0.40%) in all of the treatments, and depressed around 3.3 to 27.6 times with electron donor amendment.

Totally, 459 OTUs detected were not yet unclassified, and 30.9% of them associated with the unclassified *Sphingobacteriales* WCHB1–69 clone. Almost all of the unclassified OTUs are the rare populations in the microcosms, except three unclassified *Sphingobacteriales* WCHB1–69 OTUs (5371, 5537 and 6617) and one unclassified *Proteiniborus* OTU (9046). Within these unclassified OTUs, only two OTUs (OTU5371 and OTU6703) were commonly present in all of the microcosms and around 48% of them were unique in one of the microcosms.

These results clearly indicate that during the enrichment process, exogenous electron donor amended and PBDEs would act as two important selection factors, more functional bacteria involved in PBDEs bioremediation could be induced while some indigenous oligotrophic microorganisms could be inhibited, and the PBDE-debrominating bacteria appeared to be distinctly different from those previously reported dechlorinators.

### Relationships between microbial communities and environmental parameters

In order to understand the relationships between the microbial community structures and the environmental parameters, redundancy analysis (RDA) combined with forward selection procedure and variance inflation factors was performed to select the significant environmental variables shaping the microbial community structures at different levels. The environmental data used in these analyses, such as the geochemical properties and the concentrations of PBDEs, were presented in our previous study [14]. At phylum level, the total organic carbon in sediment (S-TOC), chemical oxygen demand (COD) and the amount of deca-BDE (Deca-BDE) were detected as the top three highest loading environmental variables, which could significantly (*p* = 0.004) explain 92.0% of the total variance with 69.0% by the first axis and 28.3% by the second axis. At class level, the top three highest loading environmental variables were the amount of hexa-BDE (Hexa-BDE), the concentration of nitrate in supernate (U-nitrate), and the total organic carbon in supernate (U-TOC), which could significantly (*p* = 0.019) explain 82.9% of the total variance with 61.0% by first axis and 26.4% by second axis. When the level was brought to genus, the top three highest loading environmental variables were identified as the amount of BDE-138 (BDE138), U-nitrate and the concentration of total nitrogen in supernate (U-TN) and these three variables could significantly (*p* = 0.014) explain 82.8% of the total variance with 60.2% by the first axis and 35.0% by the second axis, while U-nitrate and the amount of BDE-32 (BDE32) and Hexa-BDE were identified as the top three highest loading environmental variables for the microbial community at OTU level and explain 72.1% of the total variance at *p* = 0.002 (Figure S2). The constrained RDA results at different microbial community levels are also summarized in Table 2. In our previous study, RDA results showed that the microbial community structure based on the DGGE profile was highly correlated with the concentration of deca-BDE, octa-BDE and total nitrogen. These results showed that the microbial community structure was highly correlated with the PBDE congener profile. The reason that different taxonomic levels were significantly explained by different environmental variables by RDA could be due to different unclassified populations contained in different taxonomic levels.

**Table 2:** Redundancy analysis of the relationship between the microbial communities and environmental parameters.

Level	The top three highest-loading environmental variables^a^			First axis explanation (%)	Second axis explanation (%)	Total explanation (%)	*F*-ratio	*p*-value
OTU	BDE32	U-nitrate	Hexa-BDE	38.1	35.1	72.1	1.723	0.002
Genus	BDE138	U-nitrate	U-TN	60.2	25.0	82.8	3.218	0.014
Family	U-TOC	S-TOC	BDE17	54.8	29.5	76.8	2.201	0.045
Class	Hexa-BDE	U-nitrate	U-TOC	61.0	26.4	82.9	3.229	0.019
Pylum	S-TOC	U-COD	Deca-BDE	69.0	28.3	92.0	7.656	0.004

a These variables were selected by forward selection based VIF with 999 Monte Carlo permutations.

In order to identify the significant microbial groups involved in PBDEs biodegradation, the correlations among the different levels of microbial community and PBDEs congener profiles were analyzed by SPSS. For the abundant (>1.0%) phylum, only *Actinobacteria* showed significantly positive correlations (*r* = 0.862, *p* = 0.027) with tri-BDE and tetra-BDE and no significant negative correlation was observed. For the rare (<1.0%) phylum, significantly positive correlations were observed between *Aquificae* and *Cyanobacteria* with penta-, octa- and nona-BDE, and *Deinococcus-Thermus* with hepta-BDE, while negative correlations were present in Candidate_division_BRC1, Candidate_division_OP9, *Chlorobi*, *Lentisphaerae* and *Verrucomicrobia*. For the class level, significantly positive correlations with PBDE congeners were *Actinobacteria*, *Aquificae*, *Bacilli*, *Caldilineae*, *Erysipelotrichi*, *SubsectionV*, *Synergistia*, *Thermales*, and the unclassified populations, while the significantly negative correlations were *Chlorobia*, *Lentisphaeria*, and RF3. Within the commonly existing genera, significantly positive correlations with PBDE congeners were *Aminiphilus*, *Papillibacter*, *Spirochaeta*, and *Bosea*, and significantly negative correlations were *Aminobacterium*, *Azomonas*, *Anaerovorax*, *Anaerophaga*, *Clostridium*, *Tissierella*, *Oxobacter*. For the commonly existing OTUs, significantly positive correlations were only detected between the less-brominated PBDE congener (tri-BDE) and *Aminobacterium* OTU680 (*r* = 0.825, *p* = 0.043) and two unclassified OTUs, unclassified *Sphingobacteriales* bacterial clone WCHB1–69 OTU5371 (*r* = 0.853, *p* = 0.031) and unclassified *Phycisphaerae* bacterial clone vadinBA30 OTU6703 (*r* = 0.831, *p* = 0.040), and no significantly negative correlation was observed. Interestingly, almost all of the populations which showed significant correlations with PBDE congeners were low abundant (<1%) in the communities. Although the known dechlorinating bacterium, *Dehalobacter*, was only detected in the microcosms enriched with methanol and ethanol in low abundances, significantly positive correlations with tri- and tetra-BDE were observed. These results suggest that some rare populations may play key role for PBDEs biodegradation and the more-toxic lesser-brominated PBDE congeners act as the main factors for selecting the PBDE-debrominating microbial community although their structures and compositions shift with the electron donor amendments.

## Discussion

Owing to the persistent hydrophobic property of PBDEs, sediments are a significant environmental receptor for these compounds. Recent studies found that PBDEs in reducing sediments can end up in various forms and produce some toxic debromination products due to microbial activity in the environment [9]. Accumulation of PBDEs in sediment was regarded as one of serious environmental problems in the world [16]. The addition of particular electron donor has been suggested as a feasible and effective *in-situ* bioremediation strategy for recalcitrant organic contaminants [33]–[34] and heavy metals [35] by stimulating specific microbial growth and promoting the transformation of chemical compounds. It is found that the amendment of BDE-153, BDE-154 [36], trichloroethene (TCE) [9] or primer compounds (e.g.,4-bromobenzoic acid) [37] could change the bacterial communities immediately and irreversibly, and improved PBDEs degradation. Although these recent PBDE biodegradation studies have promoted the implementation of *in-situ* bioremediation strategies for sediment restoration, for optimization of these processes, a more thorough understanding of the effects of electron donors on the functional microbial community structure is still necessary.

Results of the effects of five different electron donors on PBDE degradation indicated that the electron donor amendments could dramatically shift the microbial community structure and most lesser-brominated PBDE congeners were effectively degraded in all of the microcosms after 90-day incubation compared to the initial profile of PBDEs [14]. However, the information about the PBDE-degrading microbial community and their relationships with the environmental parameters is limited due to the lack of resolution in DGGE analysis and the extremely diversity and as-yet cultivated status of microorganisms. In this study, a more sensitive and high throughput microbial community profiling method, 454 Titanium pyrosequencing, combined with multivariate statistical analyses, was performed to overcome such obstacles for studying microbial communities. The high throughput sequencing results agree well with the previous DGGE results and reveal new populations, as well as their detailed relationships with the environmental parameters at different levels.

Based on the barcoded pyrosequencing results, the microbial communities involved in PBDEs transformation not only significantly shifted with the electron donor amended, but also highly related with the PBDE congener profiles. Within the ten dominant phyla, the first abundant phylum, *Proteobacteria*, decreased in the microcosms amended with electron donor, the second, fifth and seventh abundant phylum, *Bacteroidetes*, *Spirochaetes* and unclassified phylum, enriched with electron donor amendment, but only the eighth, *Actinobacteria*, was significantly positive related with the amounts of the lesser-brominated PBDE congeners, tri-BDE and tetra-BDE. *Gammaproteobacteria*, especially *Pseudomonas* spp., were also present as the dominant organisms in all of the microcosms, which were consistent with our previous DGGE results [14], but no significant correlation was observed between these organisms and the PBDE congeners. Most of the significant correlations with the PBDE congeners were also observed in those populations with low abundances, including the known dechlorinating bacterium, *Dehalobacter*. Furthermore, some unclassified members were identified as the PBDEs-degrading populations. The unclassified *Sphingobacteriales* bacterial clone WCHB1–69, which had been previously enriched in the dioxin dehalogenating communities [38], had significantly positive correlations with the PBDE congeners. The unclassified *Phycisphaerae* bacterial clone vadinBA30 was firstly detected as the member of PBDE-degrading community, although it has been identified as a anaerobic digester for wastewater treatment [39]. Interestingly, the commonly existing microbial populations increased from five OTUs to seventeen OTUs including three unclassified OTUs after enriched in the systems containing PBDEs, and most of them were fermentation organisms enriched with the electron donor amendment. These results suggest that some rare populations may play key roles in PBDEs biodegradation and more PBDE-degrading bacteria could be enriched in the PBDEs containing systems.

In summary, the results of this study provide valuable insights into the PBDEs-degrading microbial community structures and compositions, as well as their relationships with the environmental parameters. Using the high-throughput multiplex barcoded pyrosequencing approach, the microbial community structures and compositions involved in PBDEs degradation under different electron donor amending conditions were revealed. The lesser-brominated PBDE congeners would act as the important environmental parameters for shaping the microbial community structure. Further evaluation of the functional gene compositions and microbial activity involved in PBDEs transformation would provide more information for successfully designing PBDE bioremediation strategies.

## Supporting Information

Figure S1 **Biplot of PCA of the 454 sequencing data at OTU level.** The abbreviations represent the control microcosm without electron donor amendment (C), or enriched with methanol (M), ethanol (E), acetate (A), lactate (L), or pyruvate (P).
(DOC)

Figure S2 **Biplot of RDA of the 454 sequencing data at OTU level.** The abbreviations represent the control microcosm without electron donor amendment (C), or enriched with methanol (M), ethanol (E), acetate (A), lactate (L), or pyruvate (P). Three environmental parameters, the concentration of nitrate in supernate (U-nitrate), the electron conductivity in supernate (U-cond) and total organic carbon in sediment (S-TOC), were selected by forward selection based VIF with 999 Monte Carlo permutations.
(DOC)
