# Supplementary material for: Bar-Coded Pyrosequencing Reveals the Responses of PBDE-Degrading Microbial Communities to Electron Donor Amendments
Source: PLoS One. 2012 Jan 25;7(1):e30439. doi: 10.1371/journal.pone.0030439 (PMC3266264; doi:10.1371/journal.pone.0030439)
Supplement: Figure S1 — Biplot of PCA of the 454 sequencing data at OTU level. The abbreviations represent the control microcosm without electron donor amendment (C), or enriched with methanol (M), ethanol (E), acetate (A), lactate (L), or pyruvate (P). (DOC) [file pone.0030439.s001.doc]

**Supporting information**

-1.0

1.5

-1.0

1.5

M

E

A

L

P

C

27.6%

25.4%

**Figure S1** Biplot of PCA of the 454 sequencing data at OTU level. The abbreviations represent the control microcosm without electron donor amendment (C), or enriched with methanol (M), ethanol (E), acetate (A), lactate (L), or pyruvate (P).
