# Supplementary material for: Bar-Coded Pyrosequencing Reveals the Responses of PBDE-Degrading Microbial Communities to Electron Donor Amendments
Source: PLoS One. 2012 Jan 25;7(1):e30439. doi: 10.1371/journal.pone.0030439 (PMC3266264; doi:10.1371/journal.pone.0030439)
Supplement: Figure S2 — Biplot of RDA of the 454 sequencing data at OTU level. The abbreviations represent the control microcosm without electron donor amendment (C), or enriched with methanol (M), ethanol (E), acetate (A), lactate (L), or pyruvate (P). Three environmental parameters, the concentration of nitrate in supernate (U-nitrate), the electron conductivity in supernate (U-cond) and total organic carbon in sediment (S-TOC), were selected by forward selection based VIF with 999 Monte Carlo permutations. (DOC) [file pone.0030439.s002.doc]

**Supporting information**

-1.0

1.0

-1.0

1.0

**U-cond**

**U-nitrate**

**S-TOC**

M

E

A

L

P

C

35.6%

37.8%

**Figure S2** Biplot of RDA of the 454 sequencing data at OTU level. The abbreviations represent the control microcosm without electron donor amendment (C), or enriched with methanol (M), ethanol (E), acetate (A), lactate (L), or pyruvate (P). Three environmental parameters, the concentration of nitrate in supernate (U-nitrate), the electron conductivity in supernate (U-cond) and total organic carbon in sediment (S-TOC), were selected by forward selection based VIF with 999 Monte Carlo permutations.
